# Predicting invasive disease-free survival in ER-positive, HER2-negative early breast cancer using the PAM50 risk-of-recurrence score: a retrospective analysis using single-center long-term follow-up data of postmenopausal Japanese patients

**DOI:** 10.1007/s10147-024-02604-1

**Published:** 2024-08-23

**Authors:** Akane Higami, Masahiro Takada, Nobuko Kawaguchi-Sakita, Masahiro Kawashima, Kosuke Kawaguchi, Ayane Yamaguchi, Yasuhide Takeuchi, Yosuke Yamada, Masakazu Toi

**Affiliations:** 1 Department of Breast Surgery, Shiga General Hospital, Shiga, Japan; 2 Department of Breast Surgery, Kyoto University Graduate School of Medicine, Kyoto, Japan; 3 Department of Clinical Oncology, Kyoto University Hospital, Kyoto, Japan; 4 Department of Diagnostic Pathology, Kyoto University Graduate School of Medicine, Kyoto, Japan; 5 Tokyo Metropolitan Cancer and Infectious Disease Center, Komagome Hospital, Tokyo, Japan; 6 Department of Breast Surgery, Kansai Medical University, 2-3-1, Shinmachi, Hirakata, Osaka 573-1191 Japan

**Keywords:** Breast cancer, PAM50, Genomic subtyping, Japanese patients, Postmenopausal

## Abstract

**Background:**

The prognostic value of the risk-of-recurrence (ROR) score calculated using PAM50 has been validated using clinical trials and patient cohorts. This study aimed to investigate the prognostic value of the PAM50 ROR score in Japanese patients with early breast cancer using long-term follow-up data.

**Methods:**

We enrolled postmenopausal patients with ER-positive, HER2-negative, stage I–II breast cancer who had undergone surgery at the Kyoto University Hospital between 2008 and 2014. The intrinsic subtype and ROR score were calculated using PAM50. The primary endpoint was invasive disease-free survival (IDFS).

**Results:**

We enrolled 146 patients, of whom 47 (32%) patients had node-positive disease, and 36 (25%) had received neoadjuvant or adjuvant chemotherapy. The proportions of intrinsic subtypes for luminal A, luminal B, HER2-enriched, and basal-like subtypes were 67%, 27%, 3%, and 2%, respectively. The median follow-up duration was 8.4 (range 6.3–10.0) years, and 21 IDFS events were observed. Based on the ROR score, 37%, 33%, and 30% of the patients were classified as low, intermediate, and high risks, respectively. Patients in the high-risk group had a significantly worse 8-year IDFS rate than those in the low-to-intermediate-risk groups (75.1% vs. 91.6%, *p* = 0.04). The same trend was observed in patients with and without neoadjuvant or adjuvant chemotherapy.

**Conclusions:**

Using long-term follow-up data, this study showed that the ROR score can predict the prognosis of ER-positive, HER2-negative early breast cancer in Japanese postmenopausal patients. Further investigations are required to confirm the prognostic value of the ROR score in Asian populations.

**Supplementary Information:**

The online version contains supplementary material available at 10.1007/s10147-024-02604-1.

## Introduction

Advances in systemic therapy have improved the prognosis of breast cancer. Adjuvant systemic therapy should be based on the individual risk of recurrence (ROR). Traditionally, the estimation of ROR has relied on clinical and pathological factors, such as age, tumor size, lymph node involvement, histological grade, ER status, HER2 status, and proliferative markers. Recent studies and guidelines recommend the incorporation of gene expression profiling to enhance the accuracy of predicting the ROR.

The PAM50 expression panel was developed to classify breast cancer into intrinsic subtypes [1, 2]. PAM50 also yields an ROR score by combining gene expression profiles with clinical information, including histological grade, tumor size, and lymph node metastasis status. The features of the ROR score lie in its amalgamation of gene expression profiles with clinical risk factors.

The clinical utility of the ROR score was evaluated using tumor samples from 1478 patients who had participated in the Austrian Breast and Colorectal Cancer Study Group 8 (ABCSG-8) trial of adjuvant endocrine therapy for postmenopausal patients with ER-positive disease. The ROR score added a significant prognostic value to the clinical risk model. The ROR-based risk groups separated patient prognosis, with the low-risk group demonstrating a favorable outcome [3]. Patients with luminal A-type tumors showed a significantly better prognosis than those with luminal B-type tumors. Another study utilizing the Danish Breast Cancer Cooperative Group database involved 2,558 postmenopausal patients who had received 5 years of endocrine therapy [4]. Among the lymph node-positive cases, the 10-year distant metastasis recurrence rate differed significantly between the low- and high-risk groups. Similarly, even in lymph node-negative cases, the 10-year distant metastasis recurrence rate differed significantly between the low- and high-risk groups. A similar analysis was conducted in patients who had participated in the Arimidex, Tamoxifen, Alone or in Combination (ATAC) trial of anastrozole and tamoxifen for postmenopausal patients with ER-positive disease. It revealed that the ROR provided more prognostic information than the Oncotype DX Recurrence Score. Continuous ROR scores correlated with the predicted 10-year risk of distant recurrence [5]. Nevertheless, the prognostic significance of the ROR score has not been extensively examined in Asians with early breast cancer. Therefore, this study aimed to investigate the prognostic significance of the ROR score using single-center long-term follow-up cohort data of postmenopausal patients with early breast cancer in Japan.

## Patients and methods

This was a retrospective cohort study involving postmenopausal patients with stage I–II (cT1cN0 or higher), ER-positive, HER2-negative breast cancer who had undergone surgery at the Kyoto University Hospital between January 2008 and December 2014. The study protocol was approved by the Institutional Review Board of the Kyoto University Hospital. ER, PgR, and HER2 were determined according to the ASCO CAP guidelines. Menopause was self-reported or determined by a doctor, most often based on the absence of menstruation for over a year or on blood hormone levels.

A genetic analysis of PAM50 was performed using tumor tissues from surgical specimens of the target population to evaluate the intrinsic subtype and ROR score at Medbis Co., Kyoto, Japan. Clinical information, such as tumor stage, pathological diagnosis, treatment, and outcome, was extracted from medical records. Each patient’s ROR score was calculated using the test variables that include Pearson correlations with prototypical gene expression profiles for the four intrinsic subtypes (based on a 46 gene subset of the 50 genes), a proliferation score (mean expression of an 18 gene subset of the 50 genes), and pathological tumor size. The test variables are multiplied by pre-defined weights and summed to produce the ROR score according to the formula. ROR was then categorized as low, intermediate or high-risk incorporating information on the number of positive lymph nodes.

The primary endpoint was invasive disease-free survival (IDFS) rates stratified by ROR-based risk groups and intrinsic subtypes (luminal A vs. B). Secondary endpoints included distant disease-free survival (DDFS) and overall survival. In patients without lymph node metastasis, ROR scores of 0–40, 41–60, and 61–100 were classified as low-, intermediate-, and high-risk groups, respectively. In patients with lymph node metastasis, ROR scores of 0–40 and 41–100 were classified as low- and high-risk groups, respectively. Survival curves were estimated using the Kaplan–Meier method, stratified by ROR score-based risk groups and intrinsic subtypes. Survival rates 8 years postoperatively, along with 95% confidence intervals, were calculated. Statistical analyses were performed using JMP Pro version 16.1.0 (SAS Institute, Cary, NC, USA). Statistical significance was defined as a two-sided *p* value < 0.05.

## Results

A total of 150 cases were registered, of which 4 cases were excluded (3 cases for insufficient tissue samples for genetic analysis and 1 for stage IIIA). As a result, 146 were included in the analysis. Table 1 summarizes the patient characteristics and treatment histories. The median patient age was 67 (range 46–89) years. pT1 and pT2 disease were found in 58% and 41% of the patients, respectively. Lymph node metastasis was observed in 32% of the patients. Adjuvant endocrine therapy was administered to 137 (94%) patients, and neoadjuvant or adjuvant chemotherapy was administered to 36 (25%) patients. 82% of patients received endocrine therapy including aromatase inhibitors, and 20% received it for more than 5 years. About half of the patients (48%) was classified as HER-low. The distribution of intrinsic subtypes was as follows: luminal A, 98 (67%) cases; luminal B, 40 (27%) cases; HER2-enriched, five (3%) cases; and basal-like, three (2%) cases. The median ROR score was 41 (range 0–89), with 54 (37%), 48 (33%), and 44 (30%) cases classified as low, intermediate, and high risks, respectively.

**Table 1:** Patient characteristics

Factors	*n* (%)
Age (median, range)	67 (46–89)
Tumor stage	
T1a/b	11 (8)
T1c	73 (50)
T2	60 (41)
T3	2 (1)
Nodal stage	
N0	99 (68)
N1	47 (32)
Histological grade	
1	47 (32)
2	78 (53)
3	20 (14)
Not applicable	1 (1)
HER2 score	
0	76 (52)
1 + /2 +	70 (48)
Surgery	
Lumpectomy	98 (67)
Mastectomy	48 (33)
Radiation therapy	
Yes	86 (59)
No	60 (41)
Systemic therapy	
Neoadjuvant/adjuvant chemotherapy	36 (25)
Endocrine therapy	137 (94)
Tamoxifen only	11 (8)
Aromatase inhibitors included	126 (86)
≤ 5 years	109 (75)
> 5 years	29 (20)

**Fig. 1:**
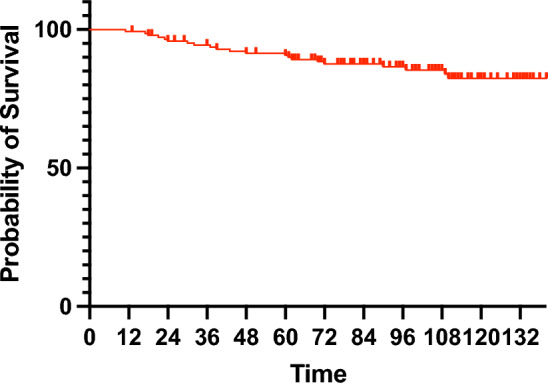
Invasive disease-free survival in an ITT population of Japanese postmenopausal patients with ER-positive breast cancer

**Fig. 2:**
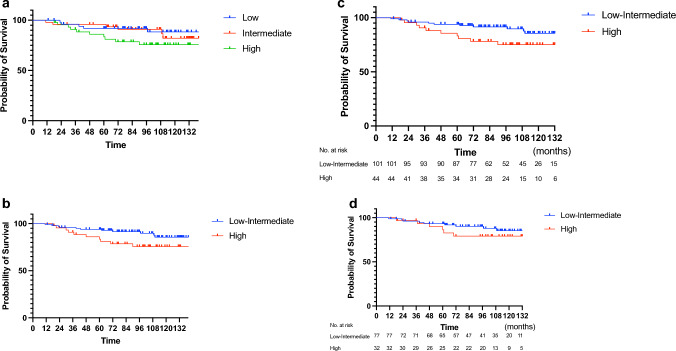
**A** Relationship between the risk of recurrence score calculated using PAM50 and invasive disease-free survival (IDFS) in Japanese postmenopausal patients with ER-positive breast cancer, divided into low-, intermediate-, and high-risk groups. **B** IDFS with separate comparisons for low-to-intermediate- and high-risk groups in all patients, **C** in patients who received neoadjuvant/adjuvant chemotherapy, **D** in patients who did not received neoadjuvant/adjuvant chemotherapy

The median follow-up duration was 8.4 (range 6.3–10.0 years), and 21 IDFS events were observed, including eight (5%) cases of local recurrence, ten (7%) cases of distant recurrence, and nine (6%) cases of death. Among the deceased patients, four (3%) were considered breast cancer-specific deaths. Four cases of recurrence occurred ≥ 5 years postoperatively. The 8-year IDFS rate was 86.4% (95% confidence interval [CI] 79.4–91.3%; Fig. 1). The 8-year IDFS rate based on the ROR score were 91.9% (95% CI 80.4–96.9%) for low risk, 91.0% (95% CI 78.1–96.6%) for intermediate risk, and 75.1% (95% CI 59.4–86.1%) for high risk (Fig. 2A). The combined 8-year IDFS rate of low- and intermediate-risk groups differed significantly from that of the high-risk group (low-to-intermediate-risk group: 91.6%, 95% CI 84.0–95.7%; high-risk group: 75.1%, 95% CI 59.4–86.1%, *p* = 0.039; Fig. 2B). In patients who had received neoadjuvant/adjuvant chemotherapy, the 8-year IDFS rate was 95.8% (95% CI 75.6–99.4%) for the low-to-intermediate-risk group and 60.0% (95% CI 27.7–85.5%) for the high-risk group (Fig. 2C). In patients who did not receive neoadjuvant/adjuvant chemotherapy, the 8-year IDFS rate was 90.2% (95% CI 80.7–95.3%) for low-to-intermediate-risk group and 79.0% (95% CI 60.5–90.3%) for the high-risk group (Fig. 2D).

**Fig. 3:**
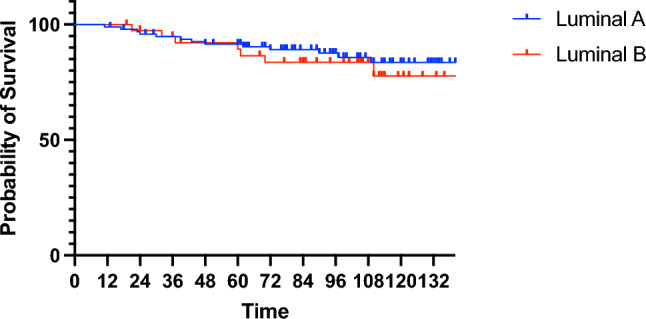
Relationship between luminal A and B subtypes by PAM50 and invasive disease-free survival in Japanese postmenopausal patients with ER-positive breast cancer

Patients with luminal A disease had a numerically higher 8-year IDFS rate compared to those with luminal B disease, although without statistical significance (87.4%, 95% CI 78.6–93.0% vs. 83.6%, 95% CI 68.0–92.5%; Fig. 3). Owing to the small number of cases and events, IDFS was not estimated for *HER2*-enriched and basal-like subtypes.

The 8-year DDFS rate was 89.9% (95% CI 83.0–94.2%; Figure S1A) overall, 95.7% (95% CI 84.5–98.9%) for the low-risk group, 92.9% (95% CI 80.2–97.7%) for the intermediate-risk group, and 79.5% (95% CI 62.6–90.0%) for the high-risk group (Figure S1B). The combined 8-year DDFS rate of low- and intermediate-risk groups differed significantly from that of the high-risk group (low-to-intermediate-risk group: 94.4%, 95% CI 87.2–97.6%; high-risk group: 79.5%, 95% CI 62.6–90.0%; *p* = 0.045; Figure S1C). The 8-year DDFS rates were 90.7% (95% CI 81.5–95.6%) for luminal A and 88.7% (95% CI 73.5–95.7%) for luminal B (Figure S1D).

Eight cases were classified as *HER2*-enriched or basal-like subtypes based on *PAM50* (Table 2). All three patients with the basal-like disease showed relatively low *ER* expressions. Of the five patients with the *HER2*-enriched disease, only one showed *HER2* expression. None of the eight patients were classified into the low-risk group.

**Table 2:** Characteristics of patients with subtypes other than the luminal type

Case	Subtype	ROR	Risk group	ER (%)	PgR (%)	HER2 score	Ki67 (%)
1	Basal-like	46	Intermediate	30	0	1 +	40
2	Basal-like	36	Intermediate	5	< 5	0	30
3	Basal-like	49	Intermediate	5	0	0	27
4	*HER2*-enriched	73	High	Allred score 3	Allred score 3	2 +	40
5	*HER2*-enriched	53	Intermediate	100	< 1	1 +	20
6	*HER2*-enriched	89	High	100	0	0	40
7	*HER2*-enriched	81	High	100	0	0	40
8	*HER2*-enriched	68	High	60	0	0	10

*ROR* rate of recurrence

We exploratory analyzed the distribution of subtype and ROR risk groups between the HER2-low and HER2-zero groups. There is no significant difference between the two groups in terms of subtype and ROR risk groups (Figure S2A). The 8-year IDFS rate of the HER2-low group (88.6%, 95%CI 77.7–94.6%) was better than that of the HER2-zero group (82.5%, 95%CI 71.5–89.8%), but the difference was not statistically different (Figure S2B).

## Discussion

We evaluated the prognostic value of the *PAM50* ROR score using long-term follow-up data from Japanese postmenopausal patients with ER-positive and HER2-negative early breast cancer. Patients classified into the high-risk group showed significantly worse IDFS than those classified into the low- or intermediate-risk groups. Most patients were classified as luminal A/B subtype, and eight (5.4%) patients were classified as *HER2*-enriched or basal-like subtypes.

The distribution of intrinsic subtypes in our cohort of patients with ER-positive and HER2-negative early breast cancer was almost consistent with that reported in previous studies. In a report by Parker et al. examining the intrinsic subtypes in patients with ER-positive breast cancer who did not receive systemic therapy, 73%, 11%, and 5% of whom were classified as luminal, HER2-enriched, and basal-like subtypes, respectively [2]. In the Trans-ATAC trial, 4.0% of patients had HER2-enriched disease, and 0.9% had basal-like disease. ABCSG-8 trial and the Danish cohort also showed similar results (supplement table). These results indicate the absence of ethnic differences in the distribution of intrinsic subtypes among patients with ER-positive, HER2-negative early breast cancer.

Our results of the relationship between prognosis and risk groups by ROR are consistent with those of previous reports (supplement table). In the ABCSG-8 trial, the luminal A type showed a significantly better prognosis compared to the luminal B type, and the low-risk group showed a favorable 10-year DDFS compared to the high-risk group. [3]. In the Danish cohort, the 10-year DDFS rate differed among risk groups. The incidence of distant recurrence was significantly lower in patients with luminal A disease than in those with luminal B disease [4]. In the present study, the high-risk group had significantly worse IDFS compared to the other groups. These results indicate that the ROR score could also provide prognostic information for Japanese postmenopausal patients with ER-positive, HER2-negative early breast cancer. In terms of the relationship between the prognosis and the intrinsic subtype, patients with luminal A- and B-type tumors showed no significant difference. Both the ABCSG8 trial and Danish cohort included patients treated with endocrine therapy alone as adjuvant treatment. In contrast, our study involved patients who received adjuvant chemotherapy in addition to adjuvant endocrine therapy based on the physician’s treatment choice. A higher proportion of patients with luminal B disease received adjuvant chemotherapy than those with luminal A disease (27.5% vs. 14.1%), which may partly explain the lack of a significant prognostic difference between luminal A and B disease.

A combined analysis of the ABCSG 8 and TransATAC trial showed that ROR scores were also useful for predicting late distant recurrence after 5 years of endocrine therapy. In the lymph node-negative/HER2-negative subgroup, ROR scores were significantly prognostic at 5–10 years. The risk of distant recurrence at 5–10 years was 16.6% (95%CI 13.1–20.9%) for the high-risk group, 8.3% (95%CI 6.1–11.2%) for the intermediate-risk group, and 2.4% (95% CI 1.6–3.5%) for the low-risk group, respectively. In our study, we were unable to examine the ability of the ROR to predict late recurrence due to the limited number of events [6].

In the present study, eight cases were classified as basal-like or HER2-enriched. These cases tended to have a higher ROR score and were classified as intermediate- or high-risk. Of these eight cases, one experienced distant recurrence and breast cancer-related death. In the present study, the basal-like type exhibited a lower rate of ER positivity. Ohara et al. reported that among patients (*n* = 16) with low ER (1–9%), 93.7% were identified as HER2-enriched or basal-like types, while only 6.3% were classified as the luminal type [7]. In another study, of all patients with ER-deficient tumors, 8% had luminal B tumors, and 48% had basal-like tumors [8]. The optimal treatment for ER-deficient tumors remains controversial and requires further investigations [9]. We also investigated the distribution of subtype and ROR risk group between HER2-low and HER2-zero but found no specific correlation.

The addition of S-1 or abemaciclib to standard endocrine therapy improved invasive disease-free survival in high risk, hormone receptor-positive, HER2-negative early breast cancer [10, 11]. Optimizing treatment requires developing biomarkers to predict the therapeutic effect of S-1 and abemaciclib. Transcriptome analysis in the monarchE trial showed a consistent benefit of adding abemaciclib to endocrine therapy across intrinsic subtypes. The relationship between the effect of S-1 and subtype or ROR has yet to be reported and needs further study.

This study had several limitations. This was a retrospective study involving a limited number of patients; therefore, the number of IDFS events was limited because we included patients with early breast cancer, which limited the statistical evaluation using the multivariate analysis. However, a strength of this study was the availability of long-term follow-up data from an academic institution, with a median follow-up duration of 8.4 years. To our knowledge, this was the first study to investigate the prognostic value of the PAM50 expression in Japanese patients with breast cancer.

The present study suggests the clinical utility of PAM50 ROR scores for predicting the prognosis in Japanese postmenopausal patients with ER-positive, HER2-negative early breast cancer, based on long-term follow-up data. The results of this study support the possibility of extrapolating previous results demonstrating the clinical utility of PAM50. Further investigations are required to confirm the prognostic value of PAM50.

## Supplementary Information

Below is the link to the electronic supplementary material.

Supplementary file1 (PDF 26 KB)

Supplementary file2 (PDF 27 KB)

Supplementary file3 (EPS 122 KB)

Supplementary file4 (EPS 118 KB)

Supplementary file5 (PDF 17 KB)

Supplementary file6 (PDF 27 KB)

Supplementary file7 (PDF 27 KB)

Supplementary file8 (PDF 25 KB)

Supplementary file9 (DOCX 18 KB)
