# Supplementary figures and images for: Predicting invasive disease-free survival in ER-positive, HER2-negative early breast cancer using the PAM50 risk-of-recurrence score: a retrospective analysis using single-center long-term follow-up data of postmenopausal Japanese patients
Source: Int J Clin Oncol. 2024 Aug 23;29(11):1715–20. doi: 10.1007/s10147-024-02604-1 (PMC11511699; doi:10.1007/s10147-024-02604-1)

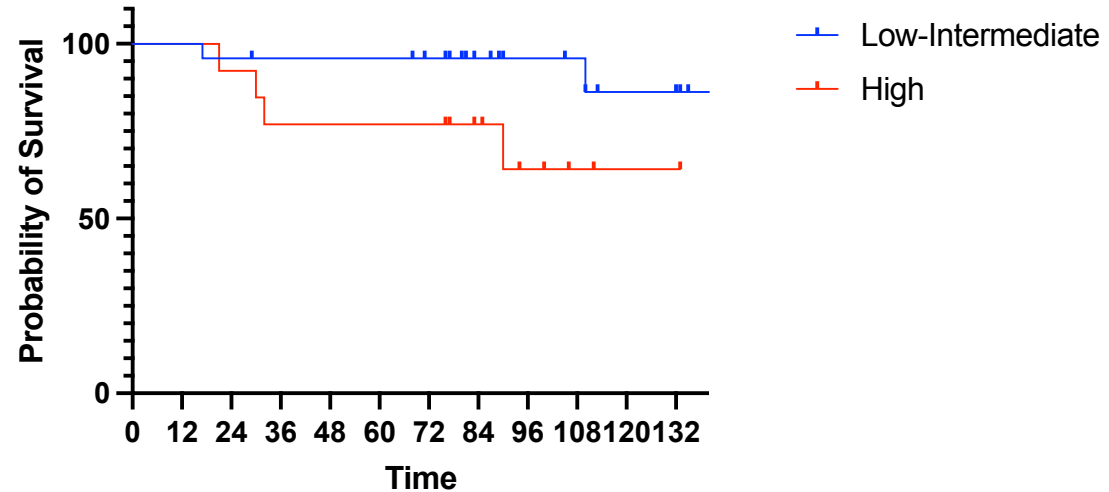

Supplement: Supplementary file 1 — Supplementary file1 (PDF 26 KB) [file 10147_2024_2604_MOESM1_ESM.pdf]

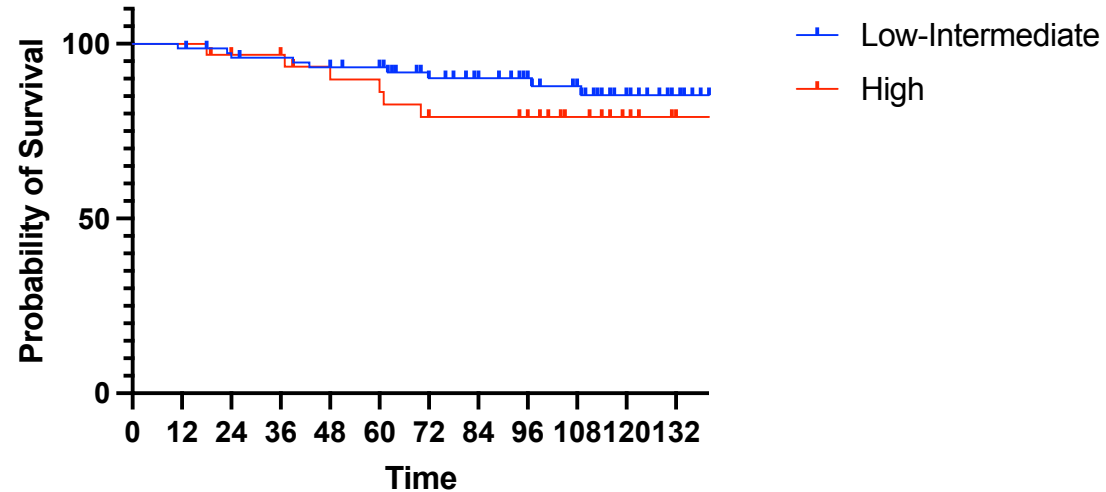

Supplement: Supplementary file 2 — Supplementary file2 (PDF 27 KB) [file 10147_2024_2604_MOESM2_ESM.pdf]

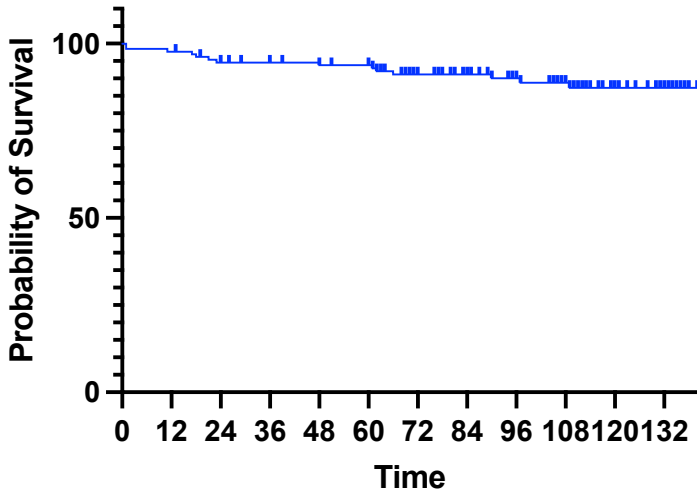

Supplement: Supplementary file 5 — Supplementary file5 (PDF 17 KB) [file 10147_2024_2604_MOESM5_ESM.pdf]

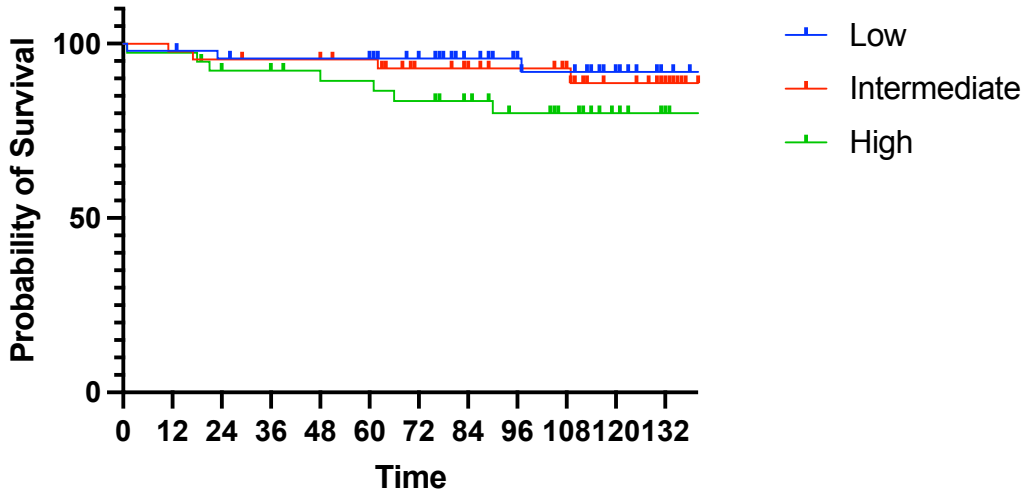

Supplement: Supplementary file 6 — Supplementary file6 (PDF 27 KB) [file 10147_2024_2604_MOESM6_ESM.pdf]

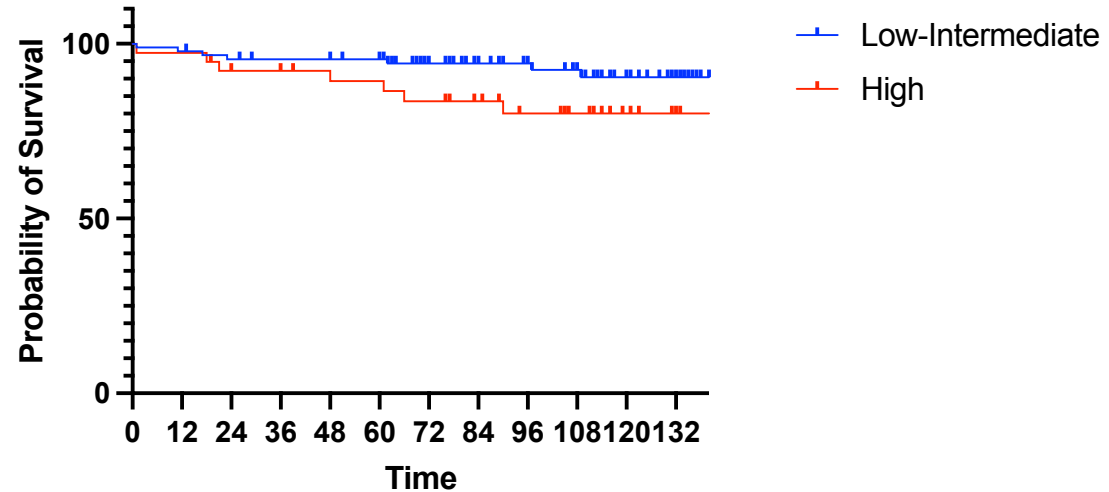

Supplement: Supplementary file 7 — Supplementary file7 (PDF 27 KB) [file 10147_2024_2604_MOESM7_ESM.pdf]

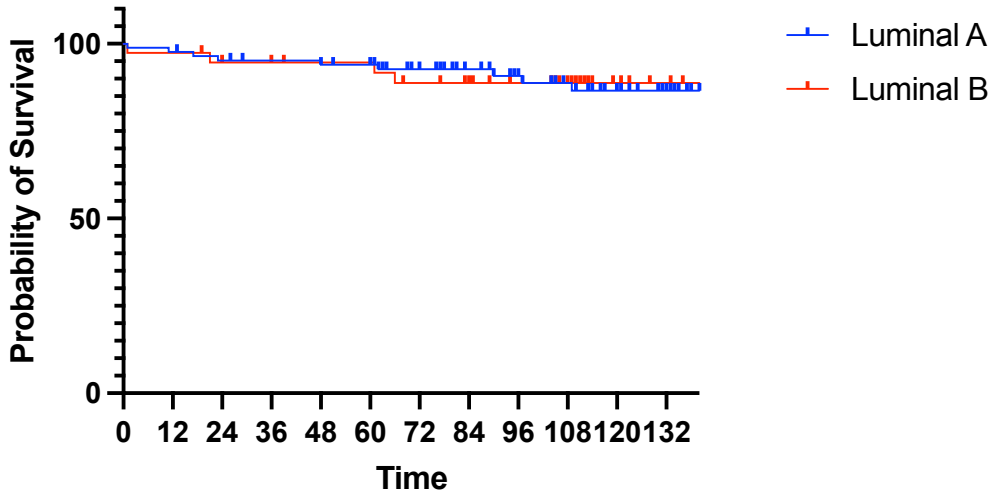

Supplement: Supplementary file 8 — Supplementary file8 (PDF 25 KB) [file 10147_2024_2604_MOESM8_ESM.pdf]
