# Supplementary material for: Predicting invasive disease-free survival in ER-positive, HER2-negative early breast cancer using the PAM50 risk-of-recurrence score: a retrospective analysis using single-center long-term follow-up data of postmenopausal Japanese patients
Source: Int J Clin Oncol. 2024 Aug 23;29(11):1715–20. doi: 10.1007/s10147-024-02604-1 (PMC11511699; doi:10.1007/s10147-024-02604-1)
Supplement: Supplementary file 9 — Supplementary file9 (DOCX 18 KB) [file 10147_2024_2604_MOESM9_ESM.docx]

**Supplement Table**

|  | **Kyoto University Hospital**  **(N=146)** | **ABCSG-8 trial**  **(N=1487)** | **Danish Cohort**  **(N=2558)** |
| --- | --- | --- | --- |
| **Age** |  |  |  |
| median (range) | 67(46-89) | 63(41-79) | 63(50-89) |
| **Tumor Stage** |  |  |  |
| T1 | 84(57.5%) | 1073(70.2%) | 1328(51.9%) |
| T2 | 60(40.8%) | 427(28.9%) |  |
| T3 | 2(1.4%) | 14(0.9%) |  |
| **Nodal Stage** |  |  |  |
| N0 | 99(67.8%) | 1047(70.8%) | 1163(45.5%) |
| N1 | 47(32.2%) | 382(25.8%) | 1395(54.5%) |
| N2 | 0% | 49(3.3%) | 0% |
| **Chemotherapy** |  |  |  |
| Yes | 36(24.7%) | 0% | 0% |
| **Intrinsic Subtype** |  |  |  |
| Luminal A type | 98(67.1%) | 1004(67.9%) | 1474(57.6%) |
| Luminal B type | 40(27.2%) | 418(28.3%) | 947(37.0%) |
| HER2-enriched type | 5(3.4%) | 48(3.2%) | 110(0.4%) |
| Basal like type | 3(2.0%) | 8(0.5%) | 27(0.1%) |
| **ROR** |  |  |  |
| Low risk | 54(36.7%) | 502(33.8%) | 720(28.1%) |
| Intermediate risk | 48(32.7%) | 478(32.1%) | 763(29.8%) |
| High risk | 44(30.1%) | 498(33.4%) | 1075（42.0%) |
|  |  |  |  |
| **Survival Outcomes** | **8y DRFS** | **10y DRFS** | **10y　DR** |
| Luminal A type | 90.8% | 93.9% | 7.6% |
| Luminal B type | 88.7% | 82.2% | 18.4% |
| ROR, low-risk | 95.7% | 96.7% | 5.0% (for N0) |
| ROR, Intermediate-risk | 92.9% | 91.3% | 7.3% (for N0) |
| ROR, High-risk | 75.5% | 79.9% | 17.8% (forN0) |

ROR, risk of recurrence score; DRFS, distant recurrence free-survival rate; DR, distant recurrence rate
